# Effect of rosiglitazone on progression of atherosclerosis: insights using 3D carotid cardiovascular magnetic resonance

**DOI:** 10.1186/1532-429X-11-24

**Published:** 2009-07-27

**Authors:** Anitha Varghese, Michael S Yee, Cheuk F Chan, Lindsey A Crowe, Niall G Keenan, Desmond G Johnston, Dudley J Pennell

**Affiliations:** 1 National Heart and Lung Institute, Imperial College, London, UK; 2 Cardiovascular Magnetic Resonance Unit, Royal Brompton Hospital, London, UK; 3 Division of Medicine, Imperial College, London, UK; 4 Department of Diabetes, Imperial College Health Care NHS Trust, London, UK

## Abstract

**Background:**

There is recent evidence suggesting that rosiglitazone increases death from cardiovascular causes. We investigated the direct effect of this drug on atheroma using 3D carotid cardiovascular magnetic resonance.

**Results:**

A randomized, placebo-controlled, double-blind study was performed to evaluate the effect of rosiglitazone treatment on carotid atherosclerosis in subjects with type 2 diabetes and coexisting vascular disease or hypertension. The primary endpoint of the study was the change from baseline to 52 weeks of carotid arterial wall volume, reflecting plaque burden, as measured by carotid cardiovascular magnetic resonance. Rosiglitazone or placebo was allocated to 28 and 29 patients respectively. Patients were managed to have equivalent glycemic control over the study period, but in fact the rosiglitazone group lowered their HbA1c by 0.88% relative to placebo (P < 0.001). Most patients received a statin or fibrate as lipid control medication (rosiglitazone 78%, controls 83%). Data are presented as mean ± SD. At baseline, the carotid arterial wall volume in the placebo group was 1146 ± 550 mm^3 ^and in the rosiglitazone group was 1354 ± 532 mm^3^. After 52 weeks, the respective volumes were 1134 ± 523 mm^3 ^and 1348 ± 531 mm^3^. These changes (-12.1 mm^3 ^and -5.7 mm^3 ^in the placebo and rosiglitazone groups, respectively) were not statistically significant between groups (P = 0.57).

**Conclusion:**

Treatment with rosiglitazone over 1 year had no effect on progression of carotid atheroma in patients with type 2 diabetes mellitus compared to placebo.

## Background

The prevalence of diabetes is increasing exponentially worldwide, and type 2 diabetes accounts for 90% of cases [1]. Insulin resistance is a fundamental feature of type 2 diabetes and is associated with increased cardiovascular risk, which accounts for up to 80% of deaths in these patients [2,3]. The United Kingdom Prospective Diabetes Study (UKPDS) demonstrated that intensive blood glucose control with insulin or sulphonylurea in type 2 diabetic patients had only a limited effect on the incidence of cardiovascular events, indicating the necessity for new treatment strategies [4].

The thiazolidinediones are a class of oral hypoglycemic drugs which have gained rapid and widespread acceptance into clinical practice. Their pharmacological action is through the reduction of insulin resistance by sensitizing muscle, liver, and adipose tissue to insulin, and treatment is associated with delayed progression to type 2 diabetes [5,6]. Their agonist effect is mediated by peroxisome proliferator activated receptor gamma (PPARγ), a nuclear hormone receptor, with effects on carbohydrate and lipid metabolism, fat cell differentiation, and gene regulation similar to those seen when insulin combines with its receptor [7]. Two glitazones are available for clinical use: pioglitazone and rosiglitazone. Both have been shown to lower hemoglobin A_1c _(HbA_1c_) to a similar extent but they have distinct lipid modulation properties [8]. Both drugs raise high-density lipoprotein cholesterol (HDLc), but pioglitazone reduces triglyceride levels while rosiglitazone has shown either no consistent change or an increase in levels. Additionally, low-density lipoprotein cholesterol (LDLc) concentration is contentious [8,9], studies have generally suggested a reduction with pioglitazone, but an increase with rosiglitazone, although the shift from small dense LDLc to a large buoyant phenotype may be less atherogenic [10].

The beneficial effect of rosiglitazone on plaque progression has been shown in animal and human subjects [11-13]. In a study of 92 sub-optimally controlled type 2 diabetic patients who were randomised to either metformin or rosiglitazone treatment, Stoker et al demonstrated that there was a significant reduction in carotid intima-media thickness (IMT) after 24 weeks in the rosiglitazone group [14]. Additionally, the effect of rosiglitazone on carotid intima-media thickness (IMT) over 12 months was studied in patients with type 2 diabetes and the insulin resistance syndrome and no statistically significant difference from placebo was shown [15]. However, this study suggested that rosiglitazone may have a beneficial effect in overt diabetes compared with the pre-diabetic group.

Carotid IMT is an important surrogate marker of cardiovascular risk and there is a linear relationship between this measure and the angiographic presence and severity of coronary artery disease [16-21]. Therapeutic intervention with antiplatelet agents, angiotensin-converting enzyme (ACE) inhibitors, β-blockers, and statins prevent progression of carotid IMT and have been shown to favorably impact on cardiovascular morbidity and mortality [22-25]. Of these, the most potent drugs are the statins [20,26].

High-resolution carotid cardiovascular magnetic resonance (CMR) is a comparatively new tool for the assessment of carotid atheroma which evaluates arterial wall remodeling in a 3-dimensional (3D) manner with good reproducibility in carotid disease of 4.4%, which allows small sample sizes [27,28]. For example, CMR showed atheroma regression using simvastatin in only 18 asymptomatic hypercholesterolaemic patients, with carotid CMR alone demonstrating a reduction of 15% in carotid vessel wall area after 1 year of statin use [14,29]. We performed a placebo-controlled, double-blind 3D carotid CMR study to evaluate the effect of rosiglitazone on atherosclerosis burden in patients with type 2 diabetes mellitus.

## Methods

This was a randomized, placebo-controlled, double-blind study in patients with type 2 diabetes and coexisting vascular disease or hypertension. After completing a 4–8 week single-blind placebo run-in period, eligible subjects entered a 52 week double-blind treatment period during which they received either rosiglitazone (4 mg once daily for the first 12 weeks and then 4 mg twice daily for the remainder of the study) or placebo. Randomization was performed in a 1:1 manner to the rosiglitazone or placebo treatment group using the Registration and Medication Ordering System (RAMOS), and stratified by statin or fibrate use without distinction between the two.

**Figure 1:**
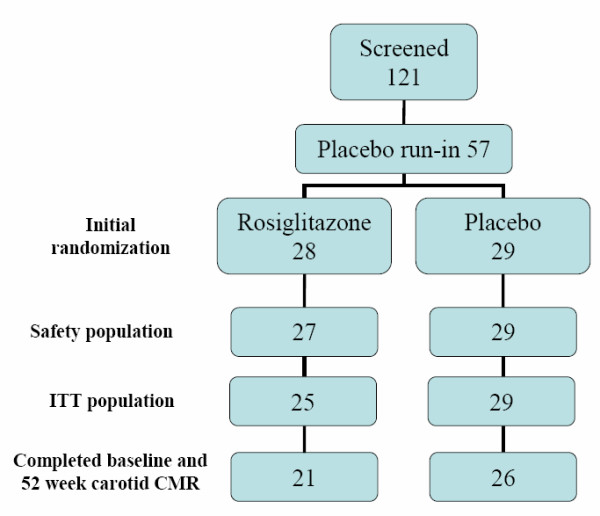
**Patient recruitment, randomization and completion**. ITT – Intention to treat.

Eligible patients were those with type 2 diabetes, aged between 30–75 years, HbA_1c _<10% at screening who had been treated with diet and exercise alone or metformin or a sulphonylurea and had been stable prior statin or fibrate dosage (for at least 3 months), and at least one atheromatous plaque causing 10–95% narrowing by ultrasound of the internal carotid artery. Exclusion criteria included more than two concomitant oral anti-hyperglycemic agents (i.e. oral combination) within 3 months of the screening visit or requirement for insulin. In total, 57 subjects were entered into the placebo run-in phase of the study and were subsequently randomized to receive double-blind medication in addition to background anti-diabetic therapy: 28 to rosiglitazone and 29 to placebo. The study protocol defined targets for glucose control during the study to achieve equivalent glycemic control between the groups. Following randomization, 3 patients did not enter the treatment phase in the rosiglitazone arm. One subject was not suitable for CMR and was not entered into the safety population. Two subjects were subsequently not entered into the intention to treat population (ITT) because of withdrawal of consent (1), and loss to follow-up (1). Twenty-one patients in the rosiglitazone group and 26 patients in the placebo group went on to complete both the baseline and 52 week CMR scans (figure 1).

The study was conducted in accordance with good clinical practice guidelines, all applicable regulatory requirements, the guiding principles of the Declaration of Helsinki, and was approved by the ethics committee. Subjects gave written informed consent. The primary endpoint of the study was total carotid atherosclerosis volume, as measured by carotid CMR arterial wall volume, following 52 weeks oral treatment with rosiglitazone compared to placebo.

Carotid CMR at baseline and week 52 was performed on a 1.5 Tesla scanner (Sonata, Siemens, Erlangen, Germany) with purpose-built bilateral four channel phased-array surface carotid coils (Machnet BV, The Netherlands), and a specially designed head and neck cushion with air-extraction for immobilization. Subjects were scanned in the supine position with the carotid coils in the magnet isocentre. T1 weighted 3D black-blood acquisitions were obtained predominantly unilaterally on the side of known carotid narrowing in all patients, and if possible bilaterally. Bilateral acquisitions were attempted if there was confirmed bilateral carotid artery disease, adequate image quality, and subjects could tolerate the additional imaging period. Typical sequence parameters were: matrix size = 256, 0.47 mm × 0.47 mm pixels; 28 slices of 2 mm thickness; typical field-of-view = 120 mm × 24 mm; time to echo = 11 ms; repetition time according to a single multiple of the subject's R-R interval; echo train length = 11; fat suppression; and 650 ms inversion time following double inversion preparation pulse during free-breathing. Acquisitions took between 2 – 4 minutes. The region chosen for all measurements were centered either side of the carotid bifurcation, extending 28 mm in both directions to give 56 mm of total vessel coverage. All scans for analysis were made perpendicular to the long-axis of the carotid artery.

**Figure 2:**
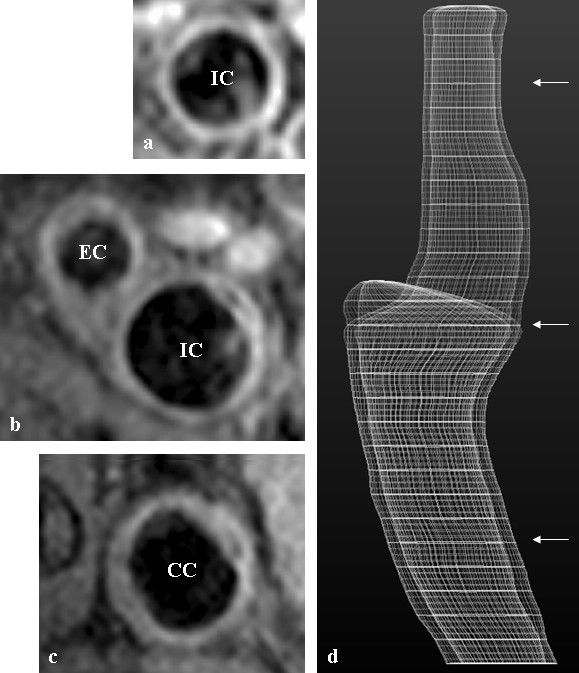
**Baseline high-resolution left carotid CMR performed on a 63 year old male study participant at the level of the a) internal carotid artery, b) carotid artery bifurcation, and c) common carotid artery, with the corresponding levels (arrowed) on the 3D model shown in d)**. CC – common carotid artery, IC – internal carotid artery, EC – external carotid artery.

The total carotid artery wall volume was calculated by subtracting total carotid luminal volume from the total vessel wall volume using semi-automated contouring software named Atheroma-Tools, (a plug-in of CMRtools, Cardiovascular Imaging Solutions, London, UK) [30]. This software models the 3D vessel with only minimal assistance from the operator (figure 2), and has been shown to facilitate greater vessel coverage than manual delineation. Contouring was performed by a single observer (AV).

Statistical analysis was performed by GlaxoSmithKline (GSK) from raw blinded information. CMR endpoints were collected separately for the left and right carotid arteries and summed for analysis where indicated. Data analysis was by ITT on the patients who were randomized, received at least one dose of medication, and had at least one post-baseline value for at least one efficacy parameter (rosiglitazone 25, placebo 29). The safety population comprised 27 patients on rosiglitazone and 29 on placebo. Summary data are presented as mean ± SD with separate calculations for each stratum within each treatment group for the total carotid wall volume at 52 weeks. The adjusted mean treatment difference between rosiglitazone and placebo is presented with a 95% two-sided CI and associated P value, with the change in total carotid wall volume from baseline to end of treatment being analyzed using parametric analysis of covariance (ANCOVA). Sample size calculations had shown that with 30 subjects per group, the study had 90% power to detect a relative effect size of 0.85.

## Results

### Study population and safety profile

The demographic profile for all subjects who had received at least one dose of study medication (safety population) is summarized in table 1, and the baseline lipid lowering and anti-hypertensive medication in table 2. At baseline, the proportion of subjects who had received previous lipid-lowering or anti-hypertensive medication was slightly lower in the rosiglitazone arm and this trend was maintained at 52 weeks. Statin use at baseline was 67% (n = 18) in the rosiglitazone group and 83% (n = 24) in the placebo group (p = 0.221). By study termination, the corresponding values were 70% (n = 19) for rosiglitazone and 93% (n = 27) for placebo (p = 0.038).

**Table 1:** Demographic characteristics of safety population

	**Rosiglitazone**	**Placebo**	**Total**	**P value**
**N (%)**	**(N = 27)**	**(N = 29)**	**(N = 56)**	
Gender				
Male	21 (78%)	23 (79%)	44 (79%)	P = 0.85
Female	6 (22%)	6 (21%)	12 (21%)	P = 0.85
Age (years)				
Mean ± SD	62.2 ± 8.2	65.6 ± 6.1	63.9 ± 7.4	P = 0.072
Median	65.0	66.0	65.0	
Range	38 – 74	55 – 75	38 – 75	
Race				
White	17 (63%)	18 (62%)	35 (63%)	P = 0.84
Black	3 (11%)	4 (14%)	7 (13%)	P = 1.00
Oriental	1 (4%)	0	1 (2%)	P = 0.48
Other	6 (22%) Asian-4, Bangladeshi-1, Brazilian-1	7 (24%) Asian-4, Bangladeshi-1, Indian-1, Yemeni-1	13 (23%)	P = 0.88
Statin/fibrate use				
Yes	21 (78%)	24 (83%)	45 (80%)	P = 0.90
No	6 (22%)	5 (17%)	11 (20%)	P = 0.89
Smoking history				
Never	6 (22%)	12 (41%)	18 (32%)	P = 0.21
Former	16 (59%)	13 (45%)	29 (52%)	P = 0.42
Current	5 (19%)	4 (14%)	9 (16%)	P = 0.73
Number of years smoked^1^				
Mean ± SD	33.5 ± 12.7	34.3 ± 14.8	33.9 ± 13.5	P = 0.83
Median	32.0	39.0	34.0	
Range	7 – 63	7 – 56	7 – 63	
Number of cigarettes per day^1^				
Mean ± SD	22.9 ± 21.5	21.7 ± 13.8	22.3 ± 18.2	P = 0.80
Median	20.0	20.0	20.0	
Range	3 – 100	3 – 60	3 – 100	
Weight (kg)				
Mean ± SD	78.1 ± 13.8	81.8 ± 14.1	80.0 ± 13.9	P = 0.59
Median	77.4	80.5	79.0	
Range	60.5 – 117.4	56.0 – 116.2	56.0 – 117.4	
Height (cm)				
Mean ± SD	167.3 ± 8.8	169.0 ± 6.2	168.2 ± 7.6	P = 0.40
Median	170.0	169.0	169.0	
Range	151 – 187	157 – 179	151 – 187	
BMI (kg/m^2^)				
Mean ± SD	27.9 ± 4.1	28.6 ± 4.3	28.3 ± 4.2	P = 0.77
Median	27.9	28.5	28.0	
Range	22.4 – 39.2	20.6 – 37.1	20.6 – 39.2	

1. Data only for current and former smokers (rosiglitazone group-21; placebo group-17).

**Table 2:** Baseline lipid-lowering and anti-hypertensive medications

	**Rosiglitazone**	**Placebo**	**P value**
**N (%)**	**(N = 27)**	**(N = 29)**	
Any such medication	26 (96%)	29 (100%)	

**Lipid-Lowering Medication**	**21 (78%)**	**24 (83%)**	**P = 0.89**
Statin	18 (67%)	24 (83%)	
Fibrate	3 (11%)	2 (7%)	
Other lipid-lowering agents	1 (4%)	1 (3%)	
			
**Anti-Hypertensive Medication**	**24 (89%)**	**29 (100%)**	**P = 0.23**
Diuretic	13 (48%)	10 (34%)	
ACE inhibitor	12 (44%)	16 (55%)	
Calcium channel antagonist	11 (41%)	19 (66%)	
Beta-blocker	9 (33%)	14 (48%)	
Alpha-blocker	4 (15%)	8 (28%)	
Angiotensin 2 antagonist	2 (7%)	3 (10%)	

NB Some subjects had been prescribed more than one lipid-lowering or anti-hypertensive medication.

A serious adverse event was defined as any event which was fatal, life threatening, disabling or incapacitating, resulted in hospitalization or prolonged a hospital stay, or was associated with a congenital abnormality or birth defect. Additionally, any event which the investigator regarded as serious or which would suggest any significant hazard, contraindication, side effect or precaution that may have been associated with the use of the drug was documented as a serious event. Two subjects in the rosiglitazone arm (7%; worsening of myocardial ischaemia and angina) and one subject in the placebo group (3%; angina) had cardiac ischemic events. There were two reports of congestive cardiac failure, both in the rosiglitazone group. Two subjects in each group had edema. There were no deaths during the study. Compliance with study tablets, measured as taking 80 – 120% of medication, was 85% for rosiglitazone and 97% for placebo (p = 0.185).

### Glycemia and lipid profile

At the end of treatment, there was a significant change from baseline in HbA_1c _in the rosiglitazone group compared with the placebo group (-0.88%, P < 0.001). The changes from baseline in lipid parameters by end of treatment are shown in table 3.

**Table 3:** Lipid profile changes in the ITT population.

**Lipid parameter**		**Rosiglitazone**	**Placebo**
**(mmol/L)**		**(N = 25)**	**(N = 29)**
**Total Cholesterol**			
Baseline	geometric mean	4.14	4.36
	(-SE, +SE)	3.97, 4.33	4.15, 4.58
End of treatment	geometric mean	4.13	4.17
	(-SE, +SE)	3.97, 4.29	3.97, 4.37
**% Change**	geometric mean	**-0.5**	**-4.5**
	(-SE, +SE)	-3.24, 2.40	-8.00, -0.85
**P value**		**0.42**	

**HDLc**			
Baseline	geometric mean	1.10	1.14
	(-SE, +SE)	1.04, 1.15	1.06, 1.22
End of treatment	geometric mean	1.16	1.22
	(-SE, +SE)	1.09, 1.22	1.15, 1.29
**% Change**	geometric mean	**5.4**	**7.2**
	(-SE, +SE)	1.41, 9.55	3.35, 11.1
**P value**		**0.97**	

**LDLc**			
Baseline	geometric mean	2.22	2.31
	(-SE, +SE)	2.07, 2.37	2.16, 2.47
End of treatment	geometric mean	2.23	2.13
	(-SE, +SE)	2.10, 2.36	1.98, 2.28
**% Change**	geometric mean	**0.4**	**-7.9**
	(-SE, +SE)	-4.07, 5.10	-12.7, -2.82
**P value**		**0.24**	

**Triglycerides**			
Baseline	geometric mean	1.61	1.67
	(-SE, +SE)	1.50, 1.72	1.52, 1.84
End of treatment	geometric mean	1.35	1.48
	(-SE, +SE)	1.24, 1.47	1.36, 1.61
**% Change**	geometric mean	**-16.0**	**-11.6**
	(-SE, +SE)	-22.4, -9.13	-18.1, -4.65
**P value**		**0.70**	

**Free fatty acids**			
Baseline	geometric mean	0.58	0.56
	(-SE, +SE)	0.55, 0.62	0.53, 0.59
End of treatment	geometric mean	0.46	0.60
	(-SE, +SE)	0.42, 0.50	0.57, 0.64
**% Change**	geometric mean	**-20.7**	**7.8**
	(-SE, +SE)	-28.00, -12.6	2.86, 12.9
**P value**		**0.0050**	

There were no significant between groups differences

% Change based on log-transformed data: 100* [exp (mean change on log scale) – 1].

### Changes in total carotid arterial wall volume

The change in carotid arterial wall volume from baseline to week 52 is summarized in table 4. At baseline, the carotid wall volume in the placebo group was 1146 ± 550 mm^3 ^and in the rosiglitazone group was 1354 ± 532 mm^3^. After 52 weeks, the respective carotid wall volumes were 1134 ± 523 mm^3 ^and 1348 ± 531 mm^3^, which was a mean decrease from baseline of 12.1 mm^3 ^and 5.7 mm^3^. These changes were small (<1%) and not statistically significant between groups (P = 0.57).

**Table 4:** Carotid CMR total wall volume changes in the ITT population

	**Treatment Group**	
	
**Week 52**	**Rosiglitazone**	**Placebo**
**(mm^3^)**	**(N = 25)**	**(N = 29)**
Number with baseline and week 52 CMR scans	21	26
Baseline (mean ± SD)	1354 ± 532	1146 ± 550
End of treatment (mean ± SD)	1348 ± 531	1134 ± 523
**Change from baseline**		
**mean ± SD**	**-5.7 ± 79.4**	**-12.1 ± 104.6**
Model adjusted change from baseline^1^		
mean ± SE	12.7 ± 22.8	-2.9 ± 20.9
Difference from placebo^1^		
mean	15.7	-
95% CI	-39.5, 70.9	-
**P-value**	**0.57**	-

1. Adjusted for stratum + baseline value + treatment.

## Discussion

This study demonstrated that rosiglitazone had no significant effect on carotid atheroma compared with placebo over 52 weeks. This is in contrast to Stocker's study [13], where they showed a significant change in the maximal and mean carotid IMT between the rosiglitazone and metformin groups. Pioglitazone has also been shown to reduce carotid IMT, independently of glycemic control in type 2 diabetes, even over 12 weeks of treatment [31]. Beneficial effects for PPARγ agonists were also shown in rabbits using CMR of the aorta [32]. Atherosclerosis was induced by double-balloon injury and a 9 month high-cholesterol diet and the rabbits were then randomized into 5 groups: continued high-cholesterol diet, normal-chow diet, normal-chow diet plus simvastatin, normal-chow plus L-805645 (a selective PPARγ agonist), and normal-chow plus simvastatin plus L-805645. Plasma cholesterol levels remained elevated in the high-cholesterol diet group but fell to similar levels in the other groups, regardless of treatment. Normalization of lipid levels in the normal-chow group halted the atheroma progression seen in the high-cholesterol group, but did not induce regression. Regression was only achieved in the groups receiving simvastatin, with the greatest effect in the group on both drug therapies. However, use of the PPARγ agonist alone had no significant effect on atheroma reduction, but did not cause progression. These findings suggested an additive anti-atherogenic effect of a statin and PPARγ agonist in the presence of a neutral lipid profile.

Recent meta-analyses suggesting an increased risk of myocardial infarction using rosiglitazone have had a negative impact on the clinical use of thiazolidinediones in type 2 diabetes, especially rosiglitazone [33,34], despite ongoing debate and the inconclusive interim report from the Rosiglitazone Evaluated for Cardiac Outcomes and Regulation of Glycemia in Diabetes (RECORD) study [35]. Our data demonstrate that rosiglitazone had no significant effect on carotid atheroma compared with placebo over 52 weeks. These findings mirror data regarding pioglitazone as assessed by coronary intravascular ultrasound (IVUS) [36]. Therefore, there is comparable data for pioglitazone and rosiglitazone, but contradictory histopathological data for L-805645.

Individual lipid pharmacokinetics may explain these differential results between drugs [37-39]. The ADOPT study showed that rosiglitazone raised LDLc compared to metformin, necessitating a greater use of lipid-lowering therapy [40]. In our study cohort, statin use was greater in the placebo group, which may have confounded our results, given the known anti-atherosclerotic effects of statins. Measured lipid parameters showed that values for HDLc increased and triglycerides decreased in both treatment groups, but there were small decreases at week 52 in total cholesterol and LDLc in the placebo group but little change in the rosiglitazone group.

Two coronary IVUS studies are of interest with regard to our study findings [41,42]. The REVERSAL trial quantified intracoronary atheroma volume following 18 months of treatment with 40 mg pravastatin versus 80 mg of atorvastatin. The moderate lipid-lowering regimen using pravastatin led to an increase in coronary atherosclerosis, while the intensive regime with atorvastatin showed absence of plaque progression over that same time period. In the ASTEROID trial, 24 months of high intensity rosuvastatin treatment (40 mg) for 24 months was needed to demonstrate coronary plaque volume reduction. This IVUS data highlights that the absence of regression of carotid atheroma over 52 weeks using rosiglitazone in the context of established lipid-lowering management is not unexpected. Also, longer study duration with more subjects would have been optimal, with repeat carotid CMR at 24 months. Our initial power calculations required 30 subjects in each arm to identify a relatively large treatment effect. However as the carotid atheroma volume changes were smaller than predicted our study was relatively underpowered and a 24 month carotid CMR may clarify the trend noted in carotid plaque volume between placebo and drug treatment. However, our findings are in line with recent data from the APPROACH study. This randomized 672 type 2 diabetic patients to either the sulfonylurea glipizide or rosiglitazone for 18 months and evaluated coronary atheroma using IVUS. These investigators found that rosiglitazone did not lead to atheroma progression or regression compared to glipizide.

Increased plaque volume is only one component of the propensity of plaque to rupture. In vivo, serial, noninvasive carotid CMR quantification of atheroma over one year reflects changes in overall plaque burden and individual plaque constituents such as smooth muscle cells, and collagen [35,43]. More detailed plaque interrogation is possible with both IVUS and CMR, and such data would provide important additional insights into the possible increased risk [37].

## Conclusion

In conclusion, 52 weeks of treatment with rosiglitazone had no effect on progression of carotid atheroma volume in patients with type 2 diabetes mellitus compared to placebo. Increased cardiovascular risk attributed to rosiglitazone cannot simply be related to increased atherosclerotic burden, and other potential mechanisms need to be considered. The adverse effects of the thiazolidinediones need to be balanced against their benefits and there should be caution with the use of surrogate markers for hard clinical end-points [44-46].

## Competing interests

AV, MSY, CFC, LAC, NGK, DJ- The authors declare that they have no competing interests.

DJP- Consultancy Siemens, Novartis and Research Support Siemens, Novartis, Director Cardiovascular Imaging Solutions

## Authors' contributions

AV- main manuscript author, acquisition of data, data analysis. MSY-patient recruitment and coordination of study, helped to draft the manuscript, data analysis. CFC- helped to draft the manuscript, data analysis. NGK, LAC- contributed to manuscript, acquisition of data. DJ-patient recruitment, participated in the study design and coordination, helped to draft the manuscript. DJP-conceived the study, participated in the study design and coordination, helped to draft the manuscript
